# Prior Abortions and Barriers to Abortion Access Reported by Pregnant Women Veterans

**DOI:** 10.1007/s11606-022-07576-4

**Published:** 2022-08-30

**Authors:** Colleen Judge-Golden, Aimee Kroll-Desrosiers, Kristin Mattocks, Sonya Borrero

**Affiliations:** 1 University of Pittsburgh School of Medicine, Pittsburgh, PA USA; 2 Department of Obstetrics & Gynecology, Duke University School of Medicine, Durham, NC USA; 3 Veterans Affairs Central Western Massachusetts Healthcare System, Northampton, MA USA; 4 University of Massachusetts Medical School, Worcester, MA USA; 5 Center for Health Equity, Research, and Promotion (CHERP), Veterans Affairs Pittsburgh Health Care System, Pittsburgh, PA USA; 6 Center for Women’s Health Research and Innovation (CWHRI), University of Pittsburgh, Pittsburgh, PA USA

## INTRODUCTION

The Veterans Affairs (VA) Health Administration does not cover or provide abortion under any circumstances^1^—a policy more stringent than the federal standard, which allows for coverage in cases of rape, incest, or life endangerment.^2^ While Veterans obtain abortions at rates similar to women in the general US population,^3^ there is little data describing abortion experiences among Veterans. We conducted telephone surveys to learn about prior abortion experiences among pregnant Veterans using VA healthcare.

## METHODS

Between April 2020 and April 2021, we surveyed 299 pregnant Veterans enrolled in the Center for Maternal and Infant Outcomes Research in Translation (COMFORT) study about prior abortion experiences. COMFORT study methods are described in detail elsewhere.^4^ Participants were asked a series of multiple choice questions including whether they had a prior abortion, reasons for abortion, barriers to abortion, and if they ever discussed abortion with a VA provider (with free-response follow-up). Here we report descriptive findings and bivariate analyses (chi-squared, Fisher’s exact or Student’s *t*-tests) of participant characteristics by any prior abortion.

## RESULTS

**Fig. 1.**
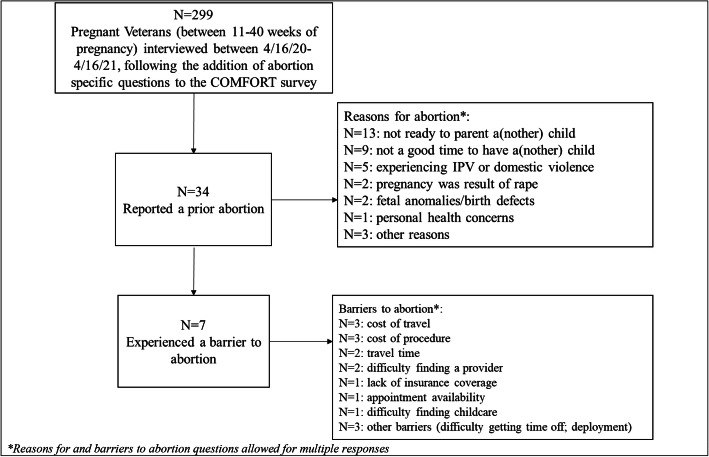
Participant flow from the COMFORT study cohort.

Overall, 34 women (11%) reported at least one prior abortion (Figure 1). Veterans reporting a prior abortion were significantly more likely to disclose history of military sexual trauma and diagnosis of post-traumatic stress disorder (Table 1).

**Table 1:** Participant Characteristics at the Time of Prenatal Telephone Survey, by Report of Prior Abortion (*N*=299)

	No prior abortion reported (*n*=265)	Prior abortion reported (*n*=34)	*P*-value*
Estimated age (mean ± SD, range)	32.2 ± 4.8 (22–44)	32.5 ± 5.0 (23–43)	0.72
Marital status (*N*, %)			0.14
Single	69 (26.0)	14 (41.2)	
Married	177 (66.8)	17 (50.0)	
Divorced/widowed/separated	18 (6.8)	3 (8.8)	
Race† (*N*, %)			0.27
White	151 (57.0)	16 (47.1)	
Black	80 (30.2)	15 (44.1)	
Asian	12 (4.5)	0 (0.0)	
Native Hawaiian or Other Pacific Islander	5 (1.9)	0 (0.0)	
American Indian or Alaska Native	5 (1.9)	1 (2.9)	
Other	40 (15.1)	6 (17.6)	
Hispanic or Latino/Latina (*N*, %)	50 (18.9)	8 (23.5)	0.54
History of military sexual trauma (*N*, %)			
Received uninvited and unwanted sexual attention while in the military	130 (49.1)	26 (76.5)	<0.01
Force or the threat of force was used to have unwanted sexual contact while in the military	74 (27.9)	21 (61.8)	<0.01
Self-reported mental health diagnoses, any time prior to survey (*N*, %)			
Depression	125 (47.2)	18 (52.9)	0.53
Post-traumatic stress disorder	91 (34.3)	19 (55.9)	0.01
Anxiety disorder	124 (46.8)	18 (52.9)	0.50

*P-values are from chi-squared or Fisher’s tests for categorical variables and Student’s *t*-test for continuous variables

†Race categories were not mutually exclusive. “Other” races included any self-specified race not fitting the categories listed

The most common reasons for seeking abortion were not being ready to parent a(nother) child (38%) and not being a good time to have a(nother) child (26%). Five women (15%) reported needing an abortion due to concurrent intimate partner violence, and two (6%) reported that the pregnancy was a result of rape. Two women reported fetal anomalies/birth defects as the reason for their abortion, and one sought abortion due to personal health problems. Seven women (21%) reported facing barriers to abortion, including cost of travel, cost of the procedure, difficulty finding a provider or appointment, travel time, and needing childcare.

Only one participant discussed their abortion with a VA provider. She explained, “the VA discouraged the decision… [my] provider only discussed negative impacts of abortion on women.”

## DISCUSSION

Greater than 10% of currently pregnant Veterans report a prior abortion, with similar reasons and barriers encountered by non-Veterans.^5,6^ Abortion is an essential component of reproductive healthcare, yet current VA policy prohibits abortion without exception.

In addition to ensuring reproductive autonomy, access to comprehensive reproductive healthcare is paramount considering that women Veterans have high rates of chronic medical and mental health conditions that may increase pregnancy-associated morbidity.^7^ Veterans who use VA healthcare are of lower socioeconomic status than Veterans with private insurance,^7^ and may therefore face greater barriers to abortion care in the private sector. Furthermore, VA serves high proportions of racial/ethnic minority women,^7^ who suffer unacceptably high rates of maternal mortality due to underlying structural and societal inequalities.^8^ While VA provides the full range of contraceptive methods at no or low-cost, pregnancy while using contraception commonly occurs and is often cited as a reason for seeking abortion.^9^

The vast majority of abortions among US women occur in free-standing clinics (i.e., not with a patient’s primary medical provider).^10^ However, while most obstetrician-gynecologists provide direct or indirect referrals for abortion,^11^ VA policy explicitly forbids abortion-related counseling in addition to abortion care and coverage.^1^ This restriction directly impedes delivery of evidence-based, quality reproductive healthcare. At a minimum, it is imperative that VA policy expand to match the standard set by other federal agencies by covering abortion in cases of rape, incest, and life endangerment. Even in our small cohort, some women sought abortions due to rape or maternal health problems. However, regardless of indication, inability to obtain a desired abortion has tremendous physical, psychological, and socioeconomic consequences,^12–14^ and even more inclusive coverage is warranted.

This study is limited by its small sample size, inclusion of only pregnant Veterans recruited via convenience sampling, and by known under-reporting of abortion leading to underestimated prevalence. Although all COMFORT participants were enrolled in VA healthcare at the time of participation, participants were not asked whether they were enrolled in VA at the time of their abortion. These issues limit the generalizability and applicability of our results. However, this exploratory study sheds light on an important and understudied issue. Our findings highlight the potential harms of current VA abortion policy, and the need for policy change.
